# Radiation-Tolerant PbS CQD Thin-Film Photodiode-Based SWIR Image Sensors

**DOI:** 10.3390/s26144404

**Published:** 2026-07-11

**Authors:** Minhyun Jin, Seungah Park, Pedro Santos, Jung-Hoon Chun, Guy Meynants, Jan Genoe, Sang Yeon Lee

**Affiliations:** 1 Interuniversity Microelectronics Centre (imec), 3001 Leuven, Belgium; minhyun.jin@imec.be (M.J.); psa8789@naver.com (S.P.); jan.genoe@imec.be (J.G.); 2 Department of Electrical and Computer Engineering, Sungkyunkwan University, Suwon 16419, Republic of Korea; jhchun@skku.edu; 3 Department of Electrical Engineering (ESAT), KU Leuven, 2440 Geel, Belgium; pedronuno.teixeiradossantos@kuleuven.be (P.S.); guy.meynants@kuleuven.be (G.M.); 4 Department of Electrical Engineering (ESAT), KU Leuven, 3001 Leuven, Belgium

**Keywords:** short-wave infrared (SWIR) image sensor, colloidal quantum dot (CQD), total ionizing dose (TID), X-ray radiation effect

## Abstract

Short-wavelength infrared (SWIR) image sensors are of increasing interest for space applications, where ionizing radiation can significantly impact device performance. PbS colloidal quantum dot (CQD)-based thin-film photodiodes (TFPDs) are promising candidates due to their spectral tunability and compatibility with CMOS integration. However, their radiation response remains insufficiently understood. We investigated the effects of X-ray irradiation on PbS CQD-based SWIR TFPDs and image sensors up to a total ionizing dose of 220 krad. The results suggest that X-ray irradiation induces ligand-dependent modulation of the trap-state in PbS CQD films, leading to reduced recombination and enhanced carrier lifetime. Consequently, the TFPDs exhibit decreased dark current and improved external quantum efficiency (EQE), reaching 44.2% at 1420 nm. PbS CQD-based SWIR image sensors maintain stable operation after irradiation until 220 krad, achieving an EQE of 33.1%. These results provide an initial assessment of PbS CQD-based SWIR image sensors under X-ray total ionizing dose (TID) exposure, highlighting the importance of ligand-dependent CQD surface chemistry towards SWIR photodetectors in space applications.

## 1. Introduction

Short-wavelength infrared (SWIR) image sensors intended for space applications must operate under harsh ionizing radiation environments, where long-term device stability and signal degradation remain critical challenges [1,2]. Since their development in the 1970s, InGaAs and HgCdTe have remained the dominant materials for SWIR detection. However, these technologies rely on expensive epitaxial growth and hybridization processes, which limit pixel scaling [3]. Thin-film photodiodes (TFPDs) based on emerging materials, such as colloidal quantum dots (CQDs), organic materials, and 2D materials, offer a promising alternative by enabling monolithic integration on complementary metal-oxide-semiconductor (CMOS) readout circuits, thereby allowing further pixel miniaturization [4,5,6,7,8,9]. Among these materials, colloidal quantum dots (CQDs) are particularly attractive for SWIR detection due to their size-tunable bandgap covering visible-to-SWIR wavelengths [10,11].

For space applications, however, SWIR image sensors must operate under harsh environments, including ionizing radiation and temperature extremes in low Earth orbit (LEO). Satellites are typically exposed to total ionizing doses (TIDs) ranging from 0.1–10 krad/year depending on orbit inclination [12,13], which can induce charge trapping and permanent degradation of device performance [14]. Nevertheless, the radiation response of CQD-based TFPDs, particularly the underlying mechanisms in the CQD layer, remains insufficiently understood. Previous studies have reported changes in CQD device performance under X-ray or gamma-ray irradiation, including variations in photocurrent and quantum efficiency [15,16]. In some cases, irradiation was reported to reduce leakage current and enhance device performance, possibly due to trap-state modification induced by radiation-generated carriers. Nevertheless, a consistent physical interpretation of these effects is still lacking [17,18].

In this work, we systematically investigate the effects of X-ray irradiation on PbS CQD-based TFPDs and image sensors under TID up to 220 krad. By correlating material-, device-, and system-level analyses, we show that X-ray irradiation response is strongly dependent on CQD ligand chemistry. The combined TRPL, C–f, C–V, dark-CELIV, dark-current, and EQE results are consistent with irradiation-induced modulation of the recombination-active trap contribution and effective charge distribution in BDT-treated PbS CQD layers. As a result, the irradiated devices exhibit improved photodetection performance, and the SWIR CQD-based image sensors maintain operation under X-ray TID up to 220 krad under the tested conditions for LEO.

## 2. X-Ray Radiation Response

### 2.1. Materials-Level Response to X-Ray Irradiation

**Figure 1:**
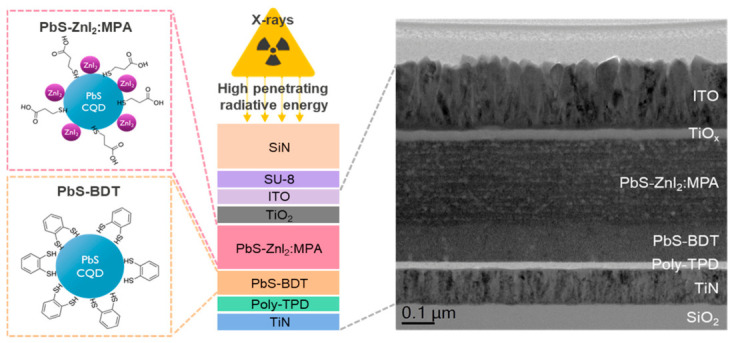
X-ray irradiation effects on CQD-TFPDs: device structure, ligand-dependent CQD behavior, radiation mechanisms, and cross-sectional HR-TEM image.

Understanding the effects of X-ray irradiation on CQD-based TFPDs and SWIR image sensors begins with the characterization of the constituent materials. Figure 1 illustrates the schematic of the X-ray irradiation test performed on the CQD-based TFPDs. The device consists of TiN as the bottom electrode, poly-TPD as the hole transport layer, PbS CQDs as the active absorber, TiO_x_ as the electron transport layer, and ITO as the top electrode. The detailed stack structure is ITO (200 nm)/TiO_2_ (20 nm)/CQD-ZnI_2_:MPA (270 nm)/CQD-BDT (110 nm)/poly-TPD (30 nm)/TiN (100 nm), as confirmed by cross-sectional high-resolution transmission electron microscopy (HR-TEM).

**Figure 2:**
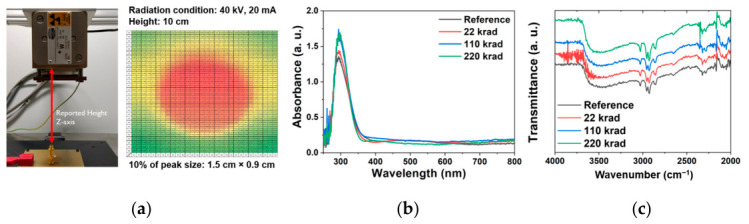
(**a**) X-ray irradiation setup and dose distribution, (**b**) TID-dependent UV–Vis–NIR absorbance of TiOx showing stable optical properties and (**c**) FT-IR spectra of poly-TPD indicating robust chemical bonding under X-ray irradiation.

Hard X-ray irradiation was performed using a tungsten Kα source with an energy of 58.7 keV, which induces ionization-related defect formation and trap states in semiconductor materials, as illustrated in Figure 2a [19]. The irradiated area was 1.5 × 0.9 cm^2^, corresponding to approximately 90% of the maximum dose uniformity. To isolate the radiation sensitivity of individual layers, each component film was independently characterized under X-ray exposure. Figure 2b shows that the TiO_2_ layer exhibits stable optical absorption under increasing TID, indicating strong robustness against X-ray irradiation. In addition, Figure 2c demonstrates that poly-TPD undergoes negligible changes in chemical bonding, as confirmed by Fourier transform infrared (FT-IR) spectroscopy, verifying the radiation stability of the organic transport layer.

Furthermore, ITO and TiN electrodes exhibit minimal variation in sheet resistance up to 220 krad, confirming the robustness of the overall charge transport framework (Table 1). These results suggest that the primary radiation-induced changes originate from the CQD active layers rather than transport or electrode layers. We attribute this behavior to ligand-dependent electronic modulation in the CQD solids. X-ray irradiation is expected to generate high-energy secondary electrons that can interact with the CQD surface, leading to modified charge distribution and an increased effective doping concentration in the CQD layer [20,21].

**Figure 3:**
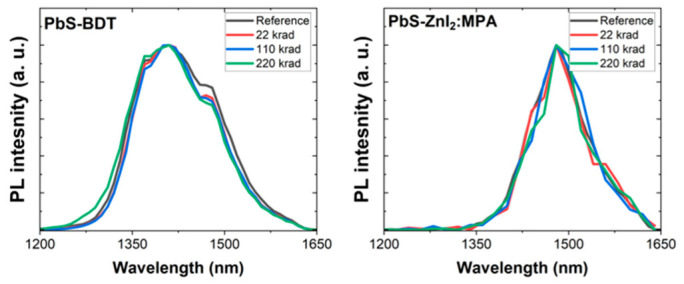
Steady-state photoluminescence (PL) spectra of CQD layers as a function of ligand type and X-ray irradiation dose, comparing PbS-BDT and PbS-ZnI_2_:MPA from non-irradiated conditions to 220 krad.

In Figure 3, steady-state photoluminescence (PL) measurements show no significant spectral shift under irradiation, indicating that the band structure remains largely unchanged.

**Figure 4:**
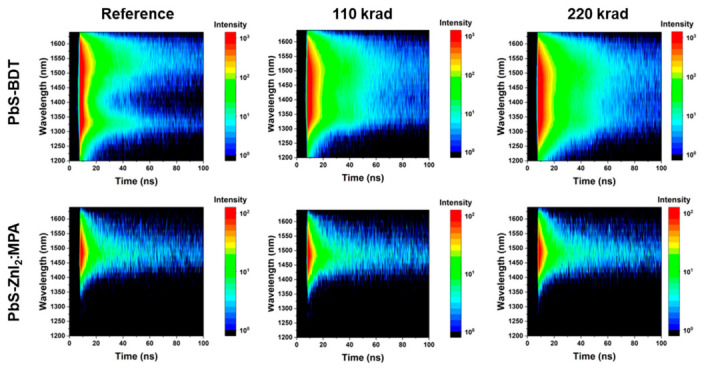
Time-resolved photoluminescence (TR-PL) contour map of CQD layers as a function of ligand type and X-ray irradiation dose, comparing PbS-BDT and PbS-ZnI_2_:MPA from non-irradiated conditions to 220 krad.

However, time-resolved photoluminescence (TRPL) measurements reveal distinct carrier dynamics depending on ligand chemistry, as shown in Figure 4. For BDT-capped CQDs, the average carrier lifetime increased from 1.56 ns before irradiation to 2.74 ns after 220 krad X-ray irradiation, while ZnI_2_:MPA-capped CQDs showed negligible variation under the same irradiation conditions [22].

**Figure 5:**
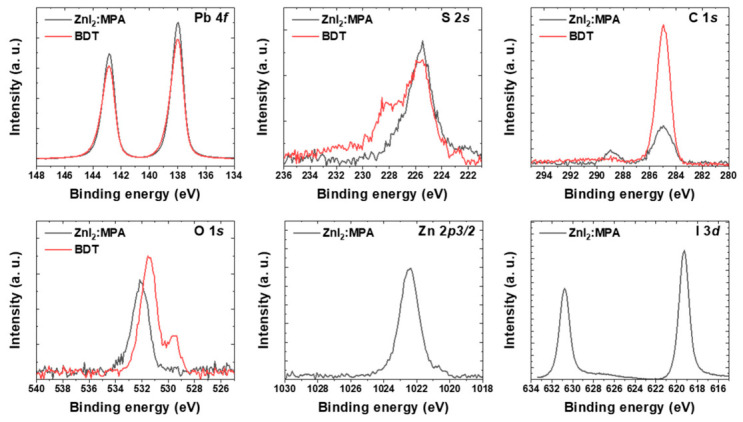
XPS spectra of PbS-BDT and PbS-ZnI_2_:MPA CQDs, showing ligand-dependent surface oxidation and chemical states.

X-ray photoelectron spectroscopy (XPS) further indicates a higher degree of surface oxidation in BDT-treated CQDs compared to ZnI_2_:MPA-treated samples, as shown in Figure 5, suggesting lower surface coverage and higher sensitivity to radiation-induced effects [22]. This difference in ligand environment leads to distinct trap-state modulation under X-ray exposure. BDT-based CQDs are more susceptible to radiation-induced modulation of surface-related electronic states, whereas ZnI_2_:MPA ligands provide a more stable surface chemistry. Overall, these results indicate that, within the ligand system investigated in this work, the X-ray radiation response of CQD films is strongly governed by ligand-dependent surface chemistry, which affects the trap-mediated charge and carrier recombination dynamics under X-ray irradiation [23].

### 2.2. Device-Level Response of CQD-Based TFPDs to X-Ray Irradiation

**Figure 6:**
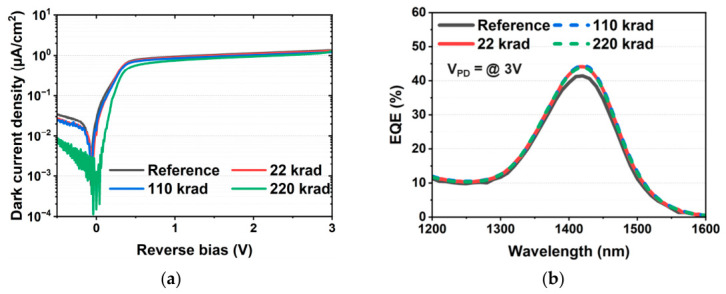
(**a**) TID-dependent dark current density of CQD-TFPDs and (**b**) EQE spectra under X-ray irradiation with improved carrier extraction.

The effects of X-ray irradiation on CQD-based TFPDs were independently investigated to evaluate device integration with Si-ROICs. Figure 6a shows the dark current density (*J_dark_*) as a function of TID. *J_dark_* decreases with increasing TID up to 110 krad and slightly increases to 220 krad, while remaining lower than that of the reference device. Figure 6b shows the EQE spectra measured at a reverse bias of 3 V. The spectral shape and peak wavelength remain unchanged across all irradiation doses, indicating that X-ray exposure does not induce significant bulk structural degradation in the CQD absorber. The EQE at 1420 nm increases from 41.6% to 44.2% with irradiation, confirming improved carrier extraction efficiency. *J_dark_* reduction should not be attributed solely to suppressed electron–hole recombination. As discussed below, dark-CELIV analysis shows that the hole mobility decreases after irradiation, which can also contribute to the lower dark current by limiting carrier transport. However, the simultaneous increase in TRPL lifetime and EQE indicates that mobility reduction alone cannot explain the overall performance trend. Therefore, *J_dark_* reduction is interpreted as a combined result of reduced recombination-active trap contribution and irradiation-induced transport modulation in the CQD layer.

**Figure 7:**
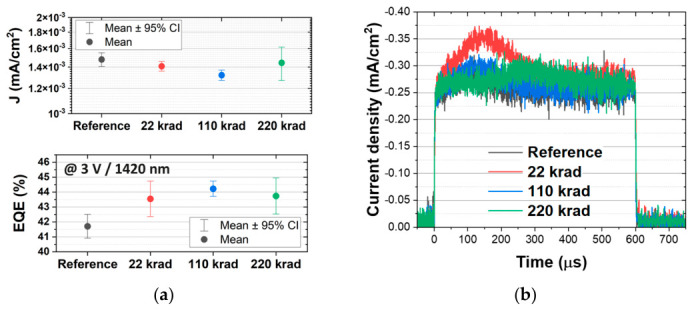
(**a**) Statistics of J_dark_ and EQE and (**b**) Dark-CELIV response under X-ray irradiation.

Figure 7a summarizes the statistical distribution of *J_dark_* and EQE for 10 devices per condition, measured at the target operating conditions of 3 V and 1420 nm, confirming consistent performance enhancement up to 110 krad followed by partial recovery at 220 krad. To further understand the device physics, dark charge extraction by linearly increasing voltage (dark-CELIV) measurements were performed, as shown in Figure 7b. The extracted parameters are summarized in Table 2, in which the displacement current (*J_disp_*) and maximum current density (*J_max_*) indicate an increase in effective carrier concentration with X-ray dose, suggesting radiation-induced modulation of doping in the CQD layers. When CQD TFPD is irradiated with X-rays at a TID of 22 krad, it shows the increased charge carrier concentration (*N_d_*) and simultaneously the decreased carrier mobility in the instance. However, as TID increases, X-ray-induced doping decreases. The dark-CELIV results indicate that irradiation affects both carrier transport and effective charge distribution in the CQD layer. Despite the increase in extraction time (*t_max_*) and the reduction in hole mobility extracted from dark-CELIV measurements, the irradiated devices exhibit enhanced EQE and suppressed dark current. This behavior indicates that the overall device performance is not governed solely by carrier transport speed but is strongly influenced by irradiation-induced changes in trap-assisted recombination dynamics.

**Figure 8:**
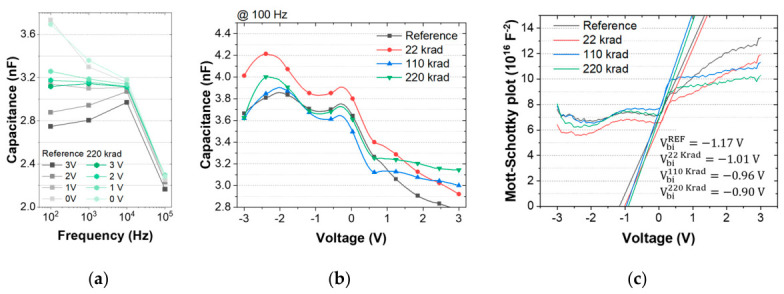
(**a**) C–f characteristics showing reduced frequency dispersion after irradiation, (**b**) C–V characteristics at 100 Hz showing stabilized depletion behavior and (**c**) Mott–Schottky analysis of depletion width with X-ray dose.

Capacitance–frequency (C–f) measurements provide an electrical probe of trap-mediated charge response in the CQD photodiodes. Electrically active trap states can exchange charge with the transport bands under low-frequency AC modulation, whereas they cannot fully follow the modulation at higher frequencies. Therefore, strong frequency dispersion in capacitance is generally associated with slow trap-mediated charge dynamics [24]. As shown in Figure 8a, the C–f dispersion is suppressed after X-ray irradiation, indicating that the contribution of electrically active trap-mediated charge response is reduced within the measured frequency window. Correspondingly, the C–V characteristics at 100 Hz show a more stable reverse-bias capacitance response after irradiation, as presented in Figure 8b. Mott–Schottky analysis further demonstrates a reduction in depletion width, as shown in Figure 8c, decreasing from 638 nm (reference) to 566 nm (22 krad), 568 nm (110 krad), and 547 nm (220 krad) [25].

**Figure 9:**
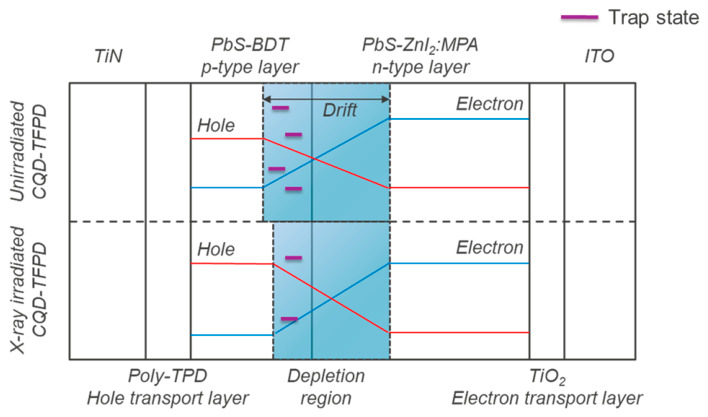
Proposed interpretation of X-ray irradiation effects, illustrating effective charge-density modulation, reduced depletion width, suppressed trap-mediated charge response, and competing transport/recombination effects in the PbS CQD TFPDs.

Although the depletion width decreases under irradiation, this reduction is not considered the direct origin of the EQE enhancement. Rather, the reduced depletion width reflects irradiation-induced modulation of the effective charge density [26]. The improved EQE is more consistently explained by reduced recombination loss under reverse bias, as supported by the increased TRPL lifetime and suppressed C–f dispersion. At the same time, dark-CELIV results indicate reduced hole mobility after irradiation. Therefore, the device response is interpreted as a balance between effective charge-density modulation, reduced recombination-active trap contribution, and mobility degradation. Figure 9 summarizes the proposed mechanism. X-ray irradiation modulates the effective carrier concentration in the PbS-BDT layer, leading to a reduced depletion width and suppressed trap-assisted recombination. Although dark-CELIV indicates mobility degradation after irradiation, the increased TRPL lifetime, suppressed C–f dispersion, and enhanced EQE suggest that reduced recombination loss improves photocarrier collection under reverse bias.

### 2.3. System-Level Radiation Hardness of CQD-Based SWIR Image Sensors

**Figure 10:**
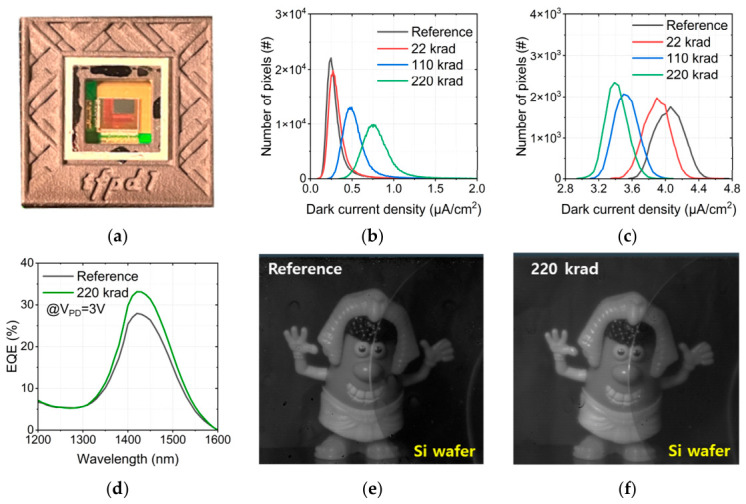
Radiation effects on the CQD-based SWIR image sensor and Si-ROIC under X-ray irradiation up to 220 krad. (**a**) Photograph of the CQD-based image sensor. (**b**) Dark current distribution of the Si-ROIC as a function of TID. (**c**) Dark current distribution of the CQD-based image sensor as a function of TID. (**d**) EQE spectra before and after irradiation. (**e**) SWIR image before irradiation. (**f**) SWIR image after irradiation.

Figure 10a shows a photograph of a CQD image sensor integrated on a Si-ROIC with a 768 × 512-pixel array and a three-transistor (3T) active pixel architecture (APS). To evaluate radiation hardness, X-rays were applied separately to the Si-ROIC and the CQD-based SWIR image sensor. X-ray irradiation was performed at room temperature up to TID of 220 krad. Figure 10b presents the dark current behavior of the Si-ROIC as a function of TID, while Figure 10c shows the corresponding dark current behavior of the CQD-based image sensor. In the case of the Si-ROIC, the peak dark current increases with increasing TID, accompanied by a broadening of the distribution, consistent with the behavior of conventional silicon-based image sensors. In contrast, the CQD-based image sensor exhibits a decrease in both peak dark current and distribution under increasing TID. This distinct behavior is consistent with the characteristics observed in CQD-based TFPDs, indicating that the dark current response to TID is strongly dependent on the CQD-based TFPD properties.

The EQE measured before and after X-ray irradiation is presented in Figure 10d. Across all irradiation doses, the spectral response remains unchanged, maintaining a peak around 1420 nm. The EQE at 1420 nm increases from 29.54% (reference) to 36.96% after 220 krad irradiation. Although the magnitude of enhancement is slightly lower than that observed in standalone TFPDs, the overall increasing trend remains consistent. The relatively lower absolute EQE values are attributed to optical losses arising from the glass encapsulation and the optical configuration of the imaging system.

In this work, the system-level evaluation was primarily focused on dark current and EQE characteristics to investigate the radiation response of the CQD-based photodetection layer. Although additional imaging parameters, including read noise, dynamic range, SNR, PRNU, and image lag, are important for evaluating practical imaging performance, their comprehensive characterization under irradiation remains for future studies.

Finally, SWIR imaging results obtained before and after irradiation are shown in Figure 10e and Figure 10f, respectively using a Si wafer as a visible-light blocking filter. These results demonstrate that the imaging performance of the system is primarily governed by the CQD-based TFPD layer rather than the underlying Si-ROIC and further confirm that stable operation is maintained even under hard X-ray radiation environments.

## 3. Experimental Environment

PbS CQD-based TFPDs were fabricated on silicon substrates with TiN bottom electrodes. Oleic acid-capped PbS CQDs (Quantum Solutions, Inc., Oxford, UK) were spin-coated on the substrates, followed by a solid-state ligand exchange process. The p-type layer was formed using 1,4-BDT, while the n-type layer was prepared using ZnI_2_ and 3-mercaptopropionic acid (MPA). A poly-TPD layer (30 nm) was spin-coated as the hole transport layer. TiOx (20 nm) was deposited by e-beam evaporation, and ITO (200 nm) was deposited by sputtering (Angstron Engineering Inc., Cambridge, Canada) as the top electrode. For image sensor fabrication, a 130-nm CMOS readout integrated circuit (ROIC) with TiN bottom contacts was used as the substrate. After integration of the CQD TFPD stack on the ROIC, photolithography and dry etching were used to define and open contact pads.

X-ray irradiation was performed using a tungsten (W) X-ray source with an energy of 58.7 keV. The TID and dose rate were calibrated in SiO_2_-equivalent units using an AVHS5 diode (CERN). The optical absorption of TiOx films was measured using a UV–vis–NIR spectrophotometer (Shimadzu Corporation, Kyoto, Japan), and the sheet resistance of electrodes was measured using a four-point probe after irradiation. The chemical properties of the poly-TPD layer were analyzed using FT-IR spectroscopy (Bruker TENSOR II, Billerica, MA, USA), while the chemical binding states of CQD layers were investigated using X-ray photoelectron spectroscopy (XPS, ULVAC-PHI VersaProbe 4, Chigasaki, Japan). Carrier dynamics were characterized using steady-state and time-resolved photoluminescence measurements (Hamamatsu Photonics, Hamamatsu, Japan).

PbS CQD-based image sensors were characterized using custom-designed readout boards. A light dome illumination system was used to acquire PTC and evaluate image lag behavior. For imaging demonstrations, an LM8HC-SW lens was attached to the sensor, and a visible-blocking setup with a lamp illumination source was used to obtain SWIR images.

## 4. Conclusions

To achieve reliable image sensors for near-Earth space applications, understanding the effects of total ionizing radiation is essential. In this work, we systematically investigated the impact of X-ray irradiation on CQD-based TFPDs from the materials to the device level. We found that X-ray irradiation primarily modulates the electronic properties of PbS-BDT CQDs, which are capped with organic ligands on the surface. In the PbS-BDT CQD layer, X-ray irradiation results in an increased carrier lifetime, suppressed capacitance–frequency dispersion, and a more stable reverse-bias C–V response. These combined optical and electrical signatures indicate a reduced contribution from electrically active trap-mediated charge dynamics and support the interpretation that recombination-active trap pathways are weakened after irradiation. As a result, the CQD TFPDs exhibit improved optoelectronic performance, including reduced dark current density and enhanced specific EQE at 1420 nm. Furthermore, these radiation-induced effects are consistently observed at the image sensor level under high TID exposure. In particular, the CQD-based image sensor demonstrates reduced dark current and enhanced EQE after irradiation, in contrast to the conventional degradation behavior of the Si-ROIC. Although this study demonstrates the TID response of PbS CQD-based SWIR TFPDs under X-ray irradiation up to 220 krad, further investigation at higher doses and under particle irradiation is required to fully evaluate their radiation tolerance in GEO and deep-space environments, where displacement damage and single-event effects may also become significant. Overall, this study provides new insight into the mechanism of radiation-induced modification in CQD photodetectors and offers a design guideline for robust CQD-based image sensors capable of operating in X-ray radiation environments.

## Figures and Tables

**Table 1:** The change in sheet resistance upon the increase in TID of X-rays.

Sheet Resistance (Ω/cm^2^)				
Electrode	Reference	22 krad	110 krad	220 krad
ITO	246.7	238.5	243.8	237.1
TiN	15.8	16.3	15.8	16.7

**Table 2:** The CQD TFPD parameters calculated from dark-CELIV measurements are according to the TID of the X-rays.

	*J_disp_* (mA/cm^2^)	*J_max_* (mA/cm^2^)	*t_max_* (µs)	*µ_h_* (10^−5^ cm^2^/Vs)	*N_d_* (10^15^/cm^3^)
Reference	0.243	0.278	106.9	3.151	2.068
22 krad	0.255	0.364	143.2	2.895	2.267
110 krad	0.251	0.299	133.9	2.128	2.136
220 krad	0.250	0.298	269.2	1.261	2.163

## Data Availability

The data presented in this study is available on request from the corresponding author.
